# The impact of prenatal alcohol exposure on gray matter volume and cortical surface area of 2 to 3‐year‐old children in a South African birth cohort

**DOI:** 10.1111/acer.14873

**Published:** 2022-05-29

**Authors:** Sivenesi Subramoney, Shantanu H. Joshi, Catherine J. Wedderburn, David Lee, Annerine Roos, Roger P. Woods, Heather J. Zar, Katherine L. Narr, Dan J. Stein, Kirsten A. Donald

**Affiliations:** ^1^ Department of Paediatrics and Child Health, Red Cross War Memorial Children's Hospital University of Cape Town Cape Town South Africa; ^2^ Department of Neurology University of California Los Angeles Los Angeles California USA; ^3^ Department of Bioengineering University of California Los Angeles Los Angeles California USA; ^4^ Department of Clinical Research London School of Hygiene and Tropical Medicine London UK; ^5^ The Neuroscience Institute University of Cape Town Cape Town South Africa; ^6^ SA MRC Unit on Risk and Resilience in Mental Disorders, Department of Psychiatry Stellenbosch University Stellenbosch South Africa; ^7^ Departments of Neurology, Psychiatry and Biobehavioral Sciences University of California Los Angeles Los Angeles California USA; ^8^ Unit on Child & Adolescent Health, South African Medical Research Council (SAMRC) University of Cape Town Cape Town South Africa; ^9^ Department of Psychiatry and Mental Health University of Cape Town Cape Town South Africa; ^10^ SU/UCT MRC Unit on Risk and Resilience in Mental Disorders University of Cape Town Cape Town South Africa

**Keywords:** brain development, fetal alcohol spectrum disorders, neuroimaging, prenatal alcohol exposure, structural MRI

## Abstract

**Background:**

There is a growing literature that demonstrates the effects of prenatal alcohol exposure (PAE) on brain development in school‐aged children. Less is known, however, on how PAE impacts the brain early in life. We investigated the effects of PAE and child sex on subcortical gray matter volume, cortical surface area (CSA), cortical volume (CV), and cortical thickness (CT) in children aged 2 to 3 years.

**Methods:**

The sample was recruited as a nested cross‐sectional substudy of the Drakenstein Child Health Study. Images from T1‐weighted magnetic resonance imaging were acquired on 47 alcohol‐exposed and 124 control children (i.e., with no or minimal alcohol exposure), aged 2 to 3 years, some of whom were scanned as neonates. Brain images were processed through automated processing pipelines using FreeSurfer version 6.0. Subcortical and *a priori* selected cortical regions of interest were compared.

**Results:**

Subcortical volume analyses revealed a PAE by child sex interaction for bilateral putamen volumes (Left: *p* = 0.02; Right: *p* = 0.01). There was no PAE by child sex interaction effect on CSA, CV, and CT. Analyses revealed an impact of PAE on CSA (*p* = 0.04) and CV (*p* = 0.04), but not CT in this age group. Of note, the inferior parietal gyrus CSA was significantly smaller in children with PAE compared to control children.

**Conclusions:**

Findings from this subgroup scanned at age 2 to 3 years build on previously described subcortical volume differences in neonates from this cohort. Findings suggest that PAE persistently affects gray matter development through the critical early years of life. The detectable influence of PAE on brain structure at this early age further highlights the importance of brain imaging studies on the impact of PAE on the young developing brain.

## Graphical abstract

There is currently limited knowledge on how PAE and sex impacts the brain during early childhood. Our findings demonstrate the influence of PAE and sex on brain structure at 2–3 years of age, suggesting that PAE persistently affects gray matter development through the critical early years of life. Our findings highlight the importance of brain imaging studies researching the impact of PAE on the young developing brain.

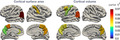

## INTRODUCTION

Fetal alcohol spectrum disorders (FASD) result from disruptions of molecular, neuroanatomical, and neurophysiological central nervous system development (Muralidharan et al., 2013; Petrelli et al., 2018). Structural magnetic resonance imaging (MRI) studies have identified overall brain volume alterations in school‐age children exposed to alcohol in‐utero in comparison with unexposed controls. The majority of findings include reports of overall volume reductions (Rajaprakash et al., 2014), although there is some evidence of volume increases (Lees et al., 2020) in children with prenatal alcohol exposure (PAE). In addition to global volume alterations, PAE may disproportionately impact specific subcortical and cortical gray matter regions. Studies have identified alterations in subcortical structures such as the hippocampi, basal ganglia, and thalami in children and adolescents with PAE (Chen et al., 2012; Nardelli et al., 2011; Zhou et al., 2018). Reports on the impact of PAE on cortical structures have typically described alterations within the frontal, temporal, and parietal lobes (Chen et al., 2012; Robertson et al., 2016; Sowell et al., 2008; Yang et al., 2012).

Specific cortical morphological differences in children with PAE have been identified in numerous indices of cortical development, including cortical surface area (CSA), cortical volume (CV), and cortical thickness (CT), across a range of ages (De Guio et al., 2014; Hendrickson et al., 2017; Rajaprakash et al., 2014; Sowell et al., 2008; Treit et al., 2013). Child age contributes to the direction of the relationship between CT and PAE. Evidence on the direction of the relationship between PAE and CT is currently mixed, however. Some studies suggest that children exposed to alcohol in‐utero have thicker cortices in comparison to control groups (Sowell, 2002; Sowell et al., 2008), whereas other studies describe thinner cortices in children with PAE in comparison with control groups (Robertson et al., 2016; Zhou et al., 2018). Recent studies suggest that CT develops in a curvilinear manner where growth peaks at 1 to 2 years and then subsequently undergoes thinning (Lyall et al., 2015; Remer et al., 2017; Wang et al., 2019). The lack of consistency in the literature may reflect samples with broad age ranges scanned at different time intervals in the course of brain development (Shaw et al., 2008). Furthermore, findings show that specific cortical morphology (e.g., CSA and CT) develop at different rates (Lyall et al., 2015) and may thus be differentially impacted by PAE (Rajaprakash et al., 2014). There is currently, however, limited knowledge on the effects of PAE on cortical gray matter development earlier in childhood. Specifically, the impact of PAE on specific morphology (i.e., CSA, CV, and CT) and the direction of the impact of PAE on cortical morphology is not known.

There is an emergent literature suggesting sex differences in the impact of PAE on brain structure in childhood. PAE has been shown to interact with sex in its impact on structural development, particularly in subcortical regions. For instance, volume alterations associated with PAE are greater in male children than female children (Inkelis et al., 2020; Nardelli et al., 2011; Treit et al., 2017). CV studies have demonstrated similar patterns of volume differences in boys but not girls with FASD, suggesting that the developmental trajectory of cortical development in children with FASD may differ according to sex (Lebel et al., 2012). This literature highlights the importance of considering potential sex differences in the impact of PAE on brain development.

The majority of published MRI studies have sampled older children, adolescents, and adults diagnosed with FASD. A few studies have demonstrated that the effects of PAE may be identified much earlier, during the neonatal period (Donald et al., 2015, 2016a, 2016b; Jacobson et al., 2017; Roos et al., 2021). However, less is known on how PAE impacts brain development in the first years of life, and how the impact of PAE on brain development may differ between boys and girls during this early period. Given that PAE may differentially impact cortical metrics (Rajaprakash et al., 2014), it is important to document the effects of PAE on both subcortical and cortical metrics during early development. The lack of studies on structural brain development among children in these first years is not limited to research on the impact of PAE on brain development. Conducting research neuroimaging on young child populations is associated with numerous practical and technical challenges (Raschle et al., 2012). The greatest gap in the imaging literature of pediatric populations is during a time when the brain undergoes rapid development and may be particularly vulnerable to the damaging effects of environmental toxins (Knickmeyer et al., 2008). Further investigation of the impact of PAE across the early developmental years is crucial to understanding how PAE may impact the neurodevelopmental trajectory.

This study was nested in the Drakenstein Child Health study (DCHS), a large South African population‐based, prospective birth cohort study investigating the early life determinants of child health (Stein et al., 2015; Zar et al., 2015). The multidisciplinary study focuses on risk factors (including PAE) and considers multiple indices of child health, including neuroimaging and developmental outcomes (Donald et al., 2018). The DCHS thus provides a unique opportunity to examine how PAE and child sex affect early brain development. We previously reported widespread structural and functional effects of PAE on a group of neonates in this cohort (Donald et al., 2015, 2016b; Roos et al., 2021) including overall gray matter volume reductions and reductions in the left hippocampus, bilateral amygdala, and left thalamus in neonates with PAE (Donald et al., 2016a). Furthermore, analysis of developmental outcomes in this cohort revealed that children exposed to PAE showed poorer motor functioning compared to control children (Hendricks et al., 2019).

In this study, we aimed to investigate the effects of PAE and child sex on structural gray matter outcomes in children between 2 and 3 years old. Specifically, we explored the effects of PAE and child sex on both total gray matter outcomes (i.e., *total* gray matter volume, subcortical volume, CSA, CV, and CV) and on *specific* subcortical and cortical regions of interest (ROIs) selected a priori. We predicted (a) persistence of some earlier‐described subcortical gray matter alterations identified in the first weeks of life at 2 to 3 years and (b) that PAE would be associated with alterations in CSA, CV, and CT ROIs that are implicated in the development of sensorimotor and frontoparietal regions in children at 2 to 3 years of age. Furthermore, we predicted that child sex would moderate the impact of PAE on subcortical and cortical ROIs.

## MATERIAL AND METHODS

### Study design

The DCHS is located in a peri‐urban area of the Western Cape, South Africa, approximately 60 km outside Cape Town. The sub‐district has a stable population of approximately 200,000, with little immigration or emigration and a well‐established primary healthcare system. Participants were recruited from two communities in the Drakenstein area, Mbekweni (serving a community with predominantly black African ancestry) and TC Newman (serving a community with predominantly mixed ancestry). Like many regions in South Africa, both communities are burdened by a high prevalence of psychosocial risk factors, including high rates of alcohol and drug use, psychological distress and depression, and exposure to violence and trauma (Donald et al., 2018; Stein et al., 2015). In addition, FASD is highly prevalent in the Western Cape region of South Africa (Olivier et al., 2016).

### Participants

#### DCHS recruitment

**FIGURE 1:**
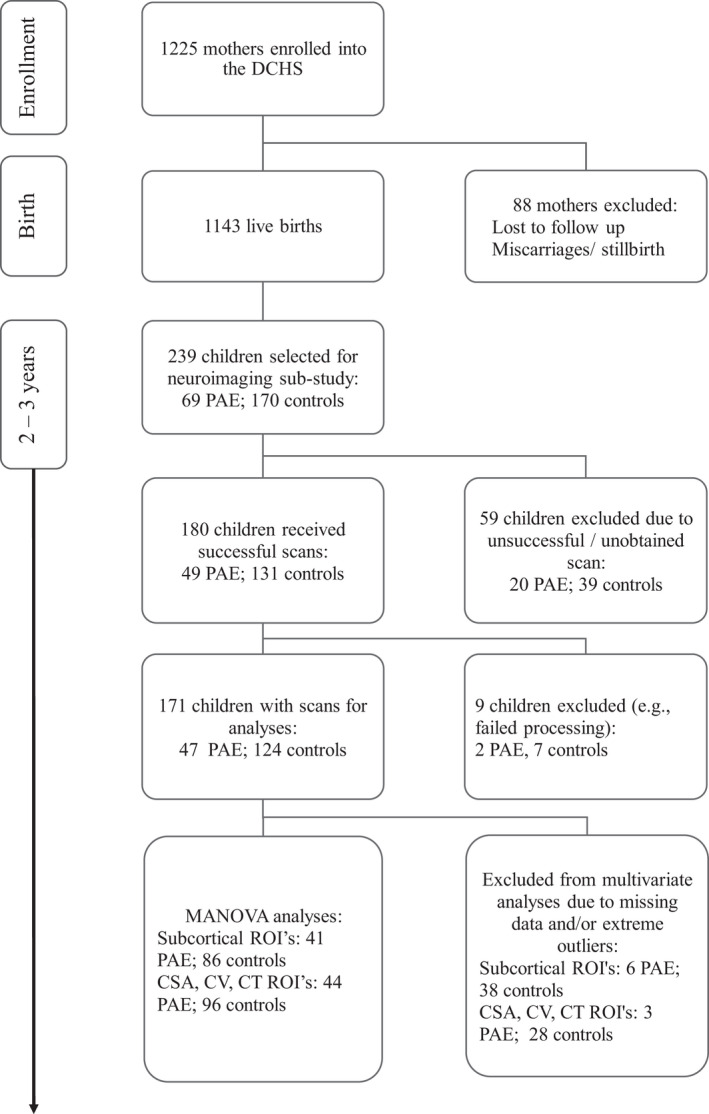
Neuroimaging in the DCHS cohort

Between March 2012 and March 2015, 1225 pregnant women were enrolled into the DCHS antenatally. Mothers were enrolled in the DCHS while attending routine antenatal care and were eligible for the study if they were 18 years or older, between 20 and 28 weeks' gestation, planned attendance at one of the two recruitment clinics, and intended to remain in the area. In total, 1137 women gave birth to 1143 live infants (four twins and one triplet). All women provided informed consent to participate in the study at enrollment (Zar et al., 2015). See Figure 1 for an outline of participant recruitment.

#### Neuroimaging sub‐study

A sub‐sample of 239 (*n* = 69 PAE; *n* = 170 controls) children from the DCHS umbrella study were included for neuroimaging at 2 to 3 years of age. The sample was comprised of children who were scanned at an earlier timepoint, as neonates (*n* = 78; *n* = 32 PAE; *n* = 46 controls) and additional children who were recruited at the 2 to 3 year timepoint. The following exclusion criteria were applied: maternal history of drug exposure on the ASSIST, prematurity (<36 weeks), low APGAR scores (<7 at 5 min), hypoxic ischemic encephalopathy, identified genetic syndrome, congenital abnormality, or admission for significant neonatal complication (Donald et al., 2016a). Mothers gave written consent at enrollment to the study and further specific consent was obtained at the neuroimaging visit from the attending parent or legal guardian. This sub‐study and the umbrella study were approved by the University of Cape Town's Human Research Ethics Committee of the Faculty of Health Sciences (HREC UCT reference 525/2012; 401/2009).

### Measures

#### Demographics

Maternal sociodemographic information was collected at 28 to 32 weeks' gestation by trained study staff. A range of sociodemographic information was collected using a standardized questionnaire, including household income, maternal age, and maternal highest level of education. Child demographic data (e.g., gestation) and health information were obtained from participants' hospital records. Details on the other psychological and child developmental measures used in the DCHS are fully described elsewhere (Donald et al., 2018; Stein et al., 2015).

#### Alcohol and substance use

Alcohol use data were collected using the prospectively administered Alcohol, Smoking, and Substance Involvement Screening Test (ASSIST) and by collecting reports of alcohol use retrospectively at the time of neonatal imaging. The ASSIST has shown good reliability and validity in international multi‐site studies (Humeniuk et al., 2008). We created a composite categorical measure (exposure vs. low exposure controls) based on antenatal ASSIST scores and more detailed quantity and timing details from the retrospective measure. Children were included in the PAE group if mothers scored a minimum of 11 on the ASSIST questionnaire during pregnancy or reported drinking (classified as consuming two or more drinks of alcohol per occasion or drinking two or more times per week) during any trimester of pregnancy retrospectively. Children whose mothers scored less than 11 on the ASSIST questionnaire were classified as low exposure controls (Donald et al., 2018). The average ASSIST score in the PAE group was 16.52 (SD = 10.92, Median = 15, IQR = 18) indicating moderate risk alcohol use. The majority of participants in the control group did not report any prenatal alcohol use. The average ASSIST score in the control group was 0.22 (SD = 0.74, Median = 0, IQR = 0) indicating low risk alcohol use. Mothers were further classified as either active smokers or nonsmokers using self‐reported smoking data obtained from the ASSIST. Mothers who reported illicit substance use on the ASSIST were excluded from participation.

#### Beck Depression Inventory

The Beck Depression Inventory (BDI‐II) was used to assess maternal symptoms of depression during pregnancy. The BDI‐II is a widely‐used and reliable measure of depressive symptoms (Beck et al., 1988) that has been used with South African populations (Stellenberg & Abrahams, 2015). The BDI‐II consists of 21 items and each item was assessed on a severity scale ranging from 0 to 3. BDI total scores were obtained by summing responses across all 21 items.

#### Image acquisition

Imaging was conducted at the Cape Town Universities Brain Imaging Centre (CUBIC). A portion of participants (*n* = 13; *n* = 11 PAE, *n* = 2 Controls) received scans on a Siemens MAGNETOM 3‐Tesla Allegra MRI system (Erlangen, Germany) at CUBIC Tygerberg Hospital. The remaining participants received scans on a Siemens MAGNETOM 3‐Tesla Skyra (Erlangen, Germany) whole body scanner at CUBIC Groote Schuur Hospital. A 32‐channel head coil was used. 3D sagittal T1‐weighted MEMPRAGE (Multi‐Echo Magnetization Prepared Rapid Acquisition Gradient Echo) images were obtained. The Siemens MAGNETOM 3‐Tesla Allegra imaging parameters were as follows: repetition time (TR) = 2530 ms; echo time (TE) = 1.53, 3.21, 4.89, 6.57 ms; flip angle = 7.0°; voxel size 1.0 × 1.0 × 1.0 mm; inversion time (TI) = 1100 ms; field of view (FOV) = 160/160/128 mm; matrix =160 × 160, 128 slices; scan time: 6 min 45 s. The Siemens MAGNETOM 3‐Tesla Skyra imaging parameters were as follows: TR = 2530 ms; TE = 1.69, 3.54, 5.39, 7.24 ms; flip angle = 7.0°; voxel size 1.0 × 1.0 × 1.0 mm; TI = 1100 ms; FOV = 224/224/176 mm; matrix = 256 × 256, 176 slices; scan time: 5 min 21 s.

#### Imaging procedure

Scanning was conducted during natural sleep at times that coincided with the children's typical sleep routine. We provided mothers and their children with door‐to‐door transport to and from CUBIC because of both the distance from home as well as the fact that many scanning slots were in the evening to coincide with bedtime. The routine to initiate sleep included a focus on creating a safe, comfortable environment, and family‐friendly approach. When the child was in a deep sleep, they were positioned in the scanner, fitted with noise‐canceling ear plugs, and their heads were stabilized with soft, foam cushions to reduce motion. A trained research assistant or research nurse remained with the child during the scan. Mothers were invited to return for a repeat scanning session if insufficient data were obtained on their first session. For detailed information on the study methods, see Wedderburn et al. (2020).

#### Image processing

Freesurfer software version 6.0 (http://www.freesurfer.net/) was used for cortical reconstruction and volume segmentation (Desikan et al., 2006). The automated processing pipeline included skull stripping, normalization and gray‐white matter segmentation, followed by surface atlas registration and data extraction. This pipeline has been documented to detect PAE effects in developing populations (Biffen et al., 2020). Cortical parcellation was conducted according to the Desikan‐Killiany atlas (Desikan et al., 2006). Output included regional brain volumes for 7 subcortical ROIs, and CSA, CV, and CT metrics for 34 ROIs. The ENIGMA pipeline protocol was followed for quality control (http://enigma.ini.usc.edu/protocols/imaging‐protocols/). Following visual inspection, extreme outliers (±3 times the IQR from the sample median) for ROI values were excluded from analyses (see Figure 1).

### Statistical analyses

Maternal and child demographic variables were expressed as mean (SD) for continuous data or absolute frequencies (%) for categorical data. Maternal and child demographic variables for children with PAE and controls were compared using *t‐*tests for continuous data and chi‐square tests for categorical data. Variables compared include child sex, gestation (weeks), maternal age at enrollment, maternal education, study site, maternal smoking status, and maternal BDI scores.

First, five separate ANOVAs were conducted to examine the effects of PAE and child sex on *total* gray matter volume, subcortical gray matter volume, CSA, CV, and CT. Models were run with and without adjustment for covariates. Second, hypothesis‐driven MANOVA analyses were then performed using ROIs selected a priori to examine the effects of PAE and child sex on regional (a) subcortical volume, (b) CSA, (c) CV, and (d) CT. Covariate‐adjusted multivariate analyses were conducted on children with complete data for all variables (See Figure 1 for details on exclusions). Subcortical regions included the left and right hemisphere of the amygdala, caudate, hippocampus, nucleus accumbens, pallidum, putamen, and thalamus (i.e., 14 subcortical ROIs were included in the model with subcortical volume as the outcome). Subcortical ROIs were selected for multivariate analyses based on prior findings described in this cohort at neonatal age (i.e., volume reductions in the left hippocampus, bilateral amygdala, and left thalamus; Donald et al., 2016a). Cortical ROIs associated with functional sensorimotor and frontoparietal networks were selected based on research indicating poorer motor outcomes on children exposed to PAE in this cohort (Hendricks et al., 2019), and early neuroimaging research indicating particularly dynamic changes within the frontoparietal network at this age (Long et al., 2017). Selected ROIs included the left and right hemisphere anterior cingulate (caudal and rostral), inferior parietal gyrus, middle frontal gyrus (caudal and rostral), paracentral gyrus, postcentral gyrus, precentral gyrus, and precuneus cortex. Thus, 18 ROIs were included in each model with CSA, CV, and CT as outcomes. Significant multivariate tests were followed‐up with post‐hoc univariate ANOVAs for each cortical ROI separately. False discovery rate (FDR) corrections were applied for the ANOVAs. Because little is known on cortical development at this timepoint, uncorrected results were also reported.

Covariates included a priori selected variables that are known to be associated with PAE (Easey et al., 2020), specifically in our cohort (Donald et al., 2016a; Hendricks et al., 2019). These variables included antenatal maternal depression (total scores), maternal education (Primary/some secondary/completed secondary), and maternal smoking status (yes/no). Given the known influence of child age on brain development at this time, we included child age at scanning as a covariate (Lyall et al., 2015). Notably, there were also small age differences between children with PAE and controls. ICV was included to correct for brain size to distinguish the effects of PAE on specific regions (Hyatt et al., 2020; Wedderburn et al., 2020). Controlling for ICV is important when examining sex differences to account for the larger overall brain volume in males (Inkelis et al., 2020). Furthermore, in the context of FASD, PAE‐related effects on specific regions may be confounded by overall brain volume reductions in exposed children (Inkelis et al., 2020; Yang et al., 2012). Finally, a small portion (*n* = 13) of the scans were conducted on a different scanner. Although steps were taken to mitigate inter‐scanner variability (e.g., the same scan sequence and data acquisition procedure was used at both sites), potential confounding related to scanner type may impact results. Scanning site was included as a covariate to minimize the potential confounding effects of the scanner on results. (Yang et al., 2012).

Additional analyses were conducted to determine the robustness of our results. MANOVAs were conducted (a) prior to covariate adjustment, (b) adjusting only for ICV, and (c) prior to adjustment for scan site. As above, significant multivariate tests were followed‐up with post‐hoc univariate ANOVAs for each cortical ROI.

All analyses were conducted using the SPSS statistical package version 27 (IBM Corp.) with *p* < 0.05 significance threshold. Partial eta‐squared values were reported to indicate effect size for ANOVA models (Richardson, 2011).

## RESULTS

### Descriptive data

Table 1 depicts the sample sociodemographic and developmental characteristics. There were no group differences in child sex, gestation, maternal age at enrollment, and monthly household income between the PAE and control children. Group differences were observed in child age at scan (*p* = 0.01), study site (*p* < 0.001), maternal active smoking during pregnancy (*p* < 0.001), maternal education (*p* = 0.01), and maternal BDI scores (*p* = 0.002). Children from the alcohol‐exposed group were younger than children from the control group. There was a higher percentage of children from the alcohol‐exposed group at TC Newman clinic than Mbekweni clinic. There was a higher prevalence of maternal active smoking and higher maternal BDI scores in the alcohol‐exposed compared with the control children, and a greater proportion of mothers with secondary and tertiary education in the control group compared with the alcohol‐exposed group.

**TABLE 1:** Description of child demographics by alcohol exposure status and infant sex

	Alcohol‐exposed group (*n* = 47)			Control group (*n* = 124)			*p*‐value
	Males (*n* = 27)	Females (*n* = 20)	Total	Males (*n* = 73)	Females (*n* = 51)	Total	
Gestation (weeks), mean (SD)	38.30 (2.05)	39.75 (1.89)	38.91 (2.09)	39.04 (2.09)	38.61 (3.13)	38.86 (2.56)	0.90
Infant age at scan (months), mean (SD)	33.59 (1.72)	32.95 (2.11)	33.32 (1.90)	34.18 (1.62)	34.24 (1.71)	34.20 (1.64)	0.01
Study site							
TC Newman, *n* (%)	16 (59.26)	12 (60.00)	28 (59.57)	17 (23.29)	10 (19.61)	27 (21.77)	<0.001
Mbekweni, *n* (%)	11 (40.74)	8 (40.00)	19 (40.43)	56 (76.71)	41 (80.39)	97 (78.23)	
Maternal age at enrollment, mean (SD)	27.35 (4.13)	27.13 (6.04)	27.26 (4.97)	28.25 (6.03)	27.02 (5.36)	27.74 (5.81)	0.61
Maternal active smoking during pregnancy, *n* (%)	18 (69.23)	11 (55.00)	29 (63.04)	16 (23.19)	14 (30.43)	30 (26.09)	<0.001
Monthly household income (ZAR), *n* (%)							
<R1000 (<75 usd)	10 (37.04)	7 (35.00)	17 (36.17)	24 (32.88)	13 (25.49)	37 (29.84)	
R1000 to R5000 (75 to 375 usd)	13 (48.15)	12 (60.00)	25 (53.19)	41 (56.16)	36 (70.59)	77 (62.10)	0.57
>R5000 (>375 usd)	4 (14.81)	1 (5.00)	5 (1.64)	8 (10.96)	2 (3.92)	10 (8.06)	
Maternal education, *n* (%)							
Primary	4 (14.81)	2 (10.00)	6 (12.77)	2 (2.74)	1 (1.96)	3 (2.42)	
Some secondary	16 (59.26)	15 (75.00)	31 (65.96)	47 (64.38)	29 (56.86)	76 (61.29)	0.01
Completed secondary and tertiary	7 (25.93)	3 (15.00)	10 (21.28)	24 (32.88)	21 (41.18)	45 (36.29)	
Maternal BDI‐II score, mean (SD)	12.24 (10.57)	14.16 (11.42)	13.07 (1.86)	7.07 (9.77)	7.51 (11.12)	7.25 (1.29)	0.002

*Note*: BDI‐II = Beck Depression Inventory. Missing data: BDI (*n* = 3 PAE; *n* = 23 controls), Smoking (*n* = 1 PAE; *n* = 9 controls).

### Total gray matter volume, subcortical volume, and CSA, volume, and thickness

Unadjusted models revealed a main effect of PAE on total gray matter volume, CSA, and CV, but not on total subcortical gray matter volume and CT (see Table 2). Children with PAE had lower total gray matter volume, CSA, and CV compared to controls. There was no effect of PAE on total gray matter volume, subcortical volume, CV, and CSA after accounting for covariates.

**TABLE 2:** Results from ANOVA on total gray matter volume, subcortical volume, CSA, CV, and CT (A) without any covariate adjustment (B) adjusting for covariates of interest

Univariate effect	ROI	Model A (unadjusted)			Model B (adjusted)		
		*F* value	Sig.	Partial eta‐squared	*F* value	Sig.	Partial eta‐squared
PAE effect	Total gray matter volume (cm^3^)	7.24	0.01	0.04	0.36	0.55	<0.001
	Total subcortical gray matter volume (cm^3^)	2.31	0.13	0.01	0.25	0.62	<0.001
	Total CSA (cm^2^)	9.41	<0.001	0.05	1.13	0.29	0.01
	Total CV (cm^3^)	8.56	<0.001	0.05	0.82	0.37	0.01
	Total CT (mm)	0.20	0.66	<0.001	<0.01	0.95	<0.001
Sex effect	Total gray matter volume (cm^3^)	41.47	<0.001	0.20	10.27	0.002	0.07
	Total subcortical gray matter volume (cm^3^)	11.77	<0.001	0.07	0.80	0.37	0.01
	Total CSA (cm^2^)	35.20	<0.001	0.17	6.09	0.01	0.04
	Total CV (cm^3^)	36.43	<0.001	0.18	6.43	0.01	0.05
	Total CT (mm)	0.04	0.84	<0.001	1.08	0.30	0.01
PAE × sex interaction effect	Total gray matter volume (cm^3^)	1.74	0.19	0.01	0.58	0.45	<0.001
	Total subcortical gray matter volume (cm^3^)	0.003	0.96	<0.001	1.45	0.23	0.01
	Total CSA (cm^2^)	2.54	0.11	0.02	2.32	0.13	0.02
	Total CV (cm^3^)	2.43	0.12	0.01	1.35	0.25	0.01
	Total CT (mm)	0.17	0.68	<0.001	0.06	0.81	<0.001

Abbreviations: CSA, cortical surface area; CT, cortical thickness; CV, cortical volume.

Unadjusted models also revealed an effect of child sex on total gray matter volume, subcortical gray matter volume, CSA, and total CV. Total gray matter volume, subcortical gray matter volume, CSA, and total CV was larger in males than females, irrespective of alcohol exposure status. The effects of child sex on total gray matter volume, CSA, and CV remained after adjusting for all covariates (see Table 3 for raw means). There was no PAE by child sex interaction effect on total gray matter volume, subcortical gray matter volume, CSA, CV, and CT either before or after covariate adjustment.

**TABLE 3:** Raw means by alcohol exposure status and infant sex for the subcortical, CSA, CV, and CT ROIs

ROI	Hemisphere	Child sex	Alcohol‐exposed group (*n* = 47)		Control group (*n* = 124)	
			Mean	SD	Mean	SD
Total gray matter volume (cm^3^)		Males	68,866.4	4746.9	69,921.7	5424.4
		Females	61,680.1	2960.5	65,379.4	4029.7
		Total	65,763.2	5404.7	68,123.7	5381.9
Total CSA (cm^2^)		Males	15,248.8	1375.4	15,538.8	1436.1
		Females	13,319.8	868.3	14,477.2	1091.8
		Total	14,415.8	1518.4	15,118.6	1405.0
Total CV (cm^3^)		Males	53,927.7	4321.0	54,823.9	4593.3
		Females	47,914.9	2533.0	51,414.9	3377.5
		Total	51,331.3	4710.0	53,474.5	4462.1
Subcortical volume ROIs (cm^3^)						
Putamen	L	Males	440.9	54.5	464.5	54.3
		Females	435.1	50.3	433.2	46.8
		Total	438.2	52.0	452.1	53.4
Putamen	R	Males	452.3	46.1	466.6	52.9
		Females	450.5	49.3	437.4	42.4
		Total	451.5	47.0	455.0	50.8
CSA ROIs (cm^2^)						
Anterior cingulate (Rostral)	L	Males	52.8	13.2	62.0	13.0
		Females	45.8	9.3	52.9	11.7
		Total	49.8	12.1	58.4	13.2
Inferior parietal	L	Males	417.2	56.1	455.2	59.7
		Females	367.7	52.3	423.1	50.0
		Total	395.8	59.3	442.5	58.0
Paracentral	L	Males	117.7	18.3	124.2	16.1
		Females	107.0	10.5	119.6	14.3
		Total	113.1	16.1	122.4	15.5
Paracentral	R	Males	130.1	18.3	135.3	16.2
		Females	116.8	11.1	131.9	17.3
		Total	124.4	16.8	134.0	16.6
Postcentral	R	Males	344.7	58.6	378.3	53.1
		Females	324.6	50.8	358.2	53.6
		Total	336.0	55.7	370.4	53.9
Precuneus	L	Males	340.9	40.2	347.0	47.9
		Females	293.1	34.2	334.6	40.0
		Total	320.3	44.3	342.1	45.1
Precuneus	R	Males	348.0	54.2	361.5	54.6
		Females	304.3	20.8	351.9	43.0
		Total	329.2	48.0	357.7	50.3
CV ROIs (cm^3^)						
Inferior parietal	L	Males	1533.4	191.9	1647.3	206.9
		Females	1370.7	204.9	1538.8	160.7
		Total	1463.1	211.6	1604.3	196.4
Middle frontal (Caudal)	L	Males	666.3	121.1	694.9	124.3
		Females	561.9	104.1	663.9	106.9
		Total	621.2	124.3	682.6	118.2
Paracentral	L	Males	375.8	61.5	398.2	50.6
		Females	342.3	48.9	381.4	55.2
		Total	361.3	58.3	391.5	52.8
Precentral	L	Males	1340.8	121.5	1402.0	166.7
		Females	1168.2	130.9	1284.3	139.8
		Total	1266.3	151.3	1355.4	166.2
Precuneus	L	Males	1153.6	141.7	1187.1	158.9
		Females	1018.6	133.1	1142.9	130.0
		Total	1095.3	152.3	1169.6	149.0
Precuneus	R	Males	1163.8	163.6	1218.6	193.8
		Females	1053.7	91.3	1198.5	145.1
		Total	1116.3	146.5	1210.6	175.6
CT ROIs (mm)						
Inferior parietal	R	Males	2.93	0.17	2.90	0.17
		Females	2.86	0.15	2.86	0.20
		Total	2.90	0.16	2.88	0.18
Postcentral	L	Males	2.44	0.17	2.48	0.17
		Females	2.44	0.18	2.39	0.14
		Total	2.44	0.17	2.45	0.16
Postcentral	R	Males	2.56	0.26	2.50	0.20
		Females	2.48	0.22	2.44	0.20
		Total	2.52	0.25	2.47	0.20

*Note*: Table depicts raw means for regions with a significant (uncorrected) main effect for PAE (CSA and CV ROIs), significant (uncorrected) main effect for sex (Total gray matter, total CSA, total CV, and CT ROIs) or a significant PAE by sex interaction (subcortical volume ROIs) on covariate adjusted models.

Abbreviations: CSA, cortical surface area; CT, cortical thickness; CV, cortical volume; ROI, region of interest.

### Subcortical ROIs analysis

The MANOVA including the subcortical ROIs (i.e., amygdala, hippocampus, thalamus, caudate, nucleus accumbens, putamen, and pallidus) revealed an overall interaction between PAE and child sex (Table 4). Follow‐up univariate analysis revealed an interaction effect for alcohol exposure and child sex on the left and the right putamen volumes (Table 5). Here, boys with PAE had smaller volumes of the left and right putamen than control boys. Girls with PAE had greater volumes of the left and right putamen than control girls (see Table 3 for raw means). There was no significant main effect for PAE or sex on subcortical volume.

**TABLE 4:** Multivariate effects for subcortical volume, CSA, CV, and CT

	Pillai's trace	*F* value	Sig.	Partial eta‐squared
Subcortical volume (cm^3^)				
PAE effect	0.17	1.49	0.13	0.17
Child sex effect	0.12	0.98	0.48	0.12
**PAE × child sex interaction effect**	**0.20**	**1.80**	**0.05**	**0.20**
CSA (cm^2^)				
**PAE effect**	**0.22**	**1.75**	**0.04**	**0.22**
Child sex effect	0.12	0.87	0.62	0.12
PAE × child sex interaction effect	0.15	1.13	0.33	0.15
CV (cm^3^)				
**PAE effect**	**0.22**	**1.74**	**0.04**	**0.22**
Child sex effect	0.16	1.22	0.26	0.16
PAE × child sex interaction effect	0.11	0.78	0.73	0.11
CT (mm)				
PAE effect	0.12	0.87	0.62	0.12
**Child sex effect**	**0.24**	**1.98**	**0.02**	**0.24**
PAE × child sex interaction effect	0.17	1.24	0.24	0.17

*Note*: Infant age at scanning, ICV, scanning site, maternal BDI scores, maternal highest level of education, and maternal smoking status were included as covariates.

Significant findings are indicated in bold.

Abbreviations: CSA, cortical surface area; CT, cortical thickness; CV, cortical volume.

**TABLE 5:** Significant follow‐up univariate effects for subcortical volume, CSA, CV, and CT adjusting for covariates of interest

ANOVA effect	ROI	Hemisphere	*F* value	Sig.	Partial eta‐squared
Subcortical PAE * sex effect	Putamen	L	5.29	0.02	0.04
	Putamen	R	8.21	0.01	0.07
CSA PAE effect (cm^2^)	Anterior cingulate (rostral)	L	4.32	0.04	0.03
	Inferior parietal	L	10.55	0.002	0.07
	Paracentral	L	6.34	0.01	0.05
	Paracentral	R	6.54	0.01	0.05
	Postcentral	R	5.90	0.02	0.04
	Precuneus	L	3.97	0.05	0.03
	Precuneus	R	6.67	0.01	0.05
CV PAE effect (cm^3^)	Inferior parietal	L	8.28	0.01	0.06
	Middle frontal (Caudal)	L	5.47	0.02	0.04
	Paracentral	L	5.03	0.03	0.04
	Precentral	L	4.21	0.04	0.03
	Precuneus	L	4.62	0.03	0.03
	Precuneus	R	6.15	0.01	0.05
CT sex effect (mm)	Inferior parietal	R	6.24	0.01	0.05
	Postcentral	L	6.73	0.01	0.05
	Postcentral	R	5.53	0.02	0.04

Abbreviations: CSA, Cortical surface area; CT, Cortical thickness; CV, cortical volume. *p*‐values are uncorrected.

### Cortical ROIs analyses

**FIGURE 2:**
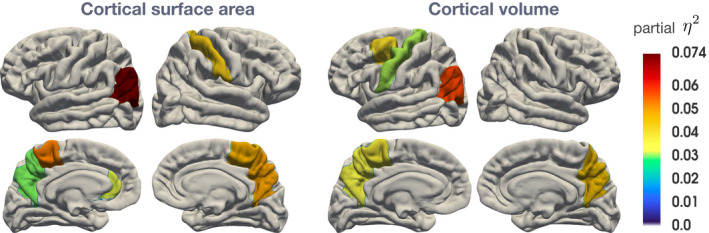
CSA and CV uncorrected ROI's with significant differences between PAE and control children (Partial Eta‐squared depicts effect size)

For CSA, the MANOVA of selected cortical ROIs revealed a significant effect of PAE. Follow‐up univariate analyses revealed CSA alterations for the left inferior parietal gyrus survived FDR correction (adjusted *p*‐value = 0.04). Univariate analyses also revealed an effect of PAE on the left rostral anterior cingulate, left and right paracentral gyrus, right postcentral gyrus, and left and right precuneus (see Figure 2). Mean CSA was lower in children with PAE compared to no‐ or minimal‐exposure children (Table 3). There was no interaction with PAE and child sex and no main effect for child sex on CSA in this cohort at this age. Statistical details for the omnibus and univariate analyses are provided in Tables 4 and 5.

The MANOVA including CV ROIs also showed a significant overall effect of PAE in the selected regions (Table 4). Univariate analyses revealed effects of child sex on the left inferior parietal gyrus, left caudal middle frontal gyrus, left paracentral gyrus, left precentral gyrus, and left and right precuneus CV (Table 5). Overall, CV was lower in children with PAE compared to controls (Table 3). There was no interaction with PAE and child sex and no main effect for child sex on CV in this cohort at this age.

The MANOVA including CT ROIs detected a significant main effect for child sex, but not for PAE (Table 4). Univariate analyses revealed effects of child sex on the left and right postcentral gyrus, and right inferior parietal CT (Table 5). Mean CT was lower in girls than in boys (Table 3). There was no interaction effect with PAE on CT. Follow‐up univariate analyses for CV and CT did not survive multiple comparison correction.

### Additional analyses

The PAE by child sex interaction on subcortical volume and PAE main effect for CSA and CV were found on the models that (a) were unadjusted for all covariates, (b) adjusted only for ICV, and (c) did not adjust for scan site (see Tables S1 to S3).

Findings pertaining to the effects of child sex differed by model, however. The model that was unadjusted for covariates revealed effects of child sex on subcortical volume, CSA, and CV, but not CT. These main effects were no longer significant when accounting for overall brain size (ICV).

## DISCUSSION

This study is the first to report the impact of PAE and sex on gray matter structure in children at 2 to 3 years of age. Hypothesis‐driven analyses revealed that areas undergoing dynamic change during this developmental period are affected by exposure to alcohol during in utero life. Child sex moderated the impact of PAE on subcortical putamen volume. In addition, specific cortical ROIs were impacted by PAE, irrespective of child sex. Specifically, ROI analyses of PAE in this cohort revealed effects on CSA and CV, but not CT in this age group.

### Effects of PAE and sex on subcortical volume

Subcortical volume findings at 2 to 3 years of age build upon the effects of PAE on subcortical brain structure identifiable during the neonatal period (Donald et al., 2016a). We found an interaction between PAE and child sex on bilateral putamen volumes, before accounting for multiple comparisons. Specifically, male children with PAE showed smaller putamen volumes compared with male control children, whereas female children with PAE did not show lower volumes compared to female control children. Previous literature has demonstrated PAE and sex interactions in children and adults (Treit et al., 2017). Although Treit et al. (2017) have previously described lower putamen volumes in both male and female children as well as adults with FASD, putamen volume alterations were greater in male children than female children. Our results demonstrate that lower putamen volume associated with PAE was evident in boys but not girls in this young cohort. Findings on control children demonstrating sex differences in putamen development are consistent with other studies on non‐clinical samples, where male children have larger putamen volumes than females (Peper et al., 2009). Findings suggest that exposure to alcohol disrupts the existing developmental sex differentiation of the putamen, resulting in male children with PAE showing smaller putamen volumes compared to female children. Our results may indicate differential putamen connectivity in boys and girls with PAE. These findings may have significant implications on developmental outcomes later in life (Cao et al., 2009; Teicher et al., 2000). For example, putamen alterations may be associated with attention and executive functioning deficits (Mattson et al., 2019). Such deficits may have long‐term implications for children with PAE.

The PAE by sex interaction effect on putamen volumes indicates that female children with PAE have larger putamen volumes than control female children. These findings are contradictory to prior literature (in a sample of children and adults) that identified smaller subcortical volume in female children with PAE compared with controls (Treit et al., 2017). Increased regional volume in children with PAE compared to controls has, however, been reported (Lees et al., 2020; Sowell, 2002) and can indicate hyperconnectivity within regions. These findings suggest that putamen developmental trajectories may be different in male children and female children with PAE. The putamen volume differences in male children and female children demonstrated in this age‐window, may reflect delayed gray matter pruning in females with PAE during a specific stage of brain maturation.

Findings from this study indicate that the effect of PAE on subcortical development identified in neonates from this cohort continued to be apparent at 2 to 3 years of age (Donald et al., 2016a). Unlike findings from the neonatal time point, we did not identify effects of PAE on the hippocampus, amygdala, and thalami as identified in neonates, after adjusting for covariates. Brain structures undergo dynamic changes from neonates to 2 to 3 years of age (Lyall et al., 2015) and cross‐sectional comparisons at different timepoints may reveal that the pattern of vulnerability to PAE may differ by timepoints. Although direct longitudinal analysis approaches including our neonate data may be possible once we acquire further imaging data at older ages, our findings allude to the importance of identifying the effects of PAE on subcortical volume across the early developmental period.

### Effects of PAE on cortical development

This study developed previous work on the impact of PAE on neonates by also investigating the effects of PAE and sex on cortical development. We found a significant multivariate effect of PAE on CSA and CV, that remained after accounting for multiple potentially confounding factors. Follow‐up analyses revealed that PAE was associated with CSA reductions in the inferior parietal gyrus in both males and females, after accounting for multiple comparisons. This finding indicates that inferior parietal CSA development may be disproportionately impacted by PAE in early life. Findings are consistent with previous studies that identified parietal lobe impact by PAE in older children (Lebel et al., 2012; Sowell et al., 2008). Uncorrected results also revealed several other fronto‐parietal (i.e., left rostral anterior, left and right precuneus for CSA and left caudal middle frontal, left inferior parietal, and left and right precuneus for CV) and motor (right postcentral gyrus, right and left paracentral gyrus for CSA and left precentral and left paracentral for CV) ROIs that were impacted by PAE in both male children and female children. These findings show similar effects of PAE on CSA and CV and suggest that the disproportionate alterations in frontal and parietal lobe volume and morphology in older age groups with PAE may be identifiable even in early childhood (Sowell et al., 2008).

Evidence suggests that cortical development is non‐uniform and that the frontal and parietal lobes undergo rapid development in the first 2 years of life (Knickmeyer et al., 2008; Lyall et al., 2015). Exposure to substances can alter the rate of dynamic change and have ongoing implications on the development of these regions outside of these critical windows (Hendrickson et al., 2018). Potential mechanisms for these findings include reduced cortical folding during gestation (Garel et al., 2003) and reduced neuronal production resulting in delayed CSA development in children with PAE.

Our multivariate results showing different effects of exposure in CSA and CV but not CT are of interest given that existing research shows that both CT and CSA contribute to CV development (Lyall et al., 2015). Findings demonstrating effects of alcohol exposure on CSA but not CT have been identified in children with alcohol‐related neurodevelopmental disorders (ARND; Rajaprakash et al., 2014). Although CT and CSA are related metrics, the two metrics show different developmental trajectories (Lyall et al., 2015). CSA development originates during embryogenesis during symmetric division of progenitor cells. CT is thought to develop later during asymmetric division of progenitor cells and cell migration (Rajaprakash et al., 2014). These differences in the development of CSA and CT also suggest that regions may show different patterns of vulnerability to alcohol exposure. For example, alcohol exposure during embryogenesis may result in greater alteration to CSA development relative to CT development.

The disproportionate impact of PAE on CSA relative to CT in this study may also be related to sample age. The majority of the existing literature on PAE and CT has been conducted on older age groups that comprise of wide age ranges. For example, studies that reported thinner or thicker cortices in children with FASD were conducted with participants in mid‐late childhood, adolescence, and adulthood (Robertson et al., 2016; Sowell, 2002; Sowell et al., 2008; Zhou et al., 2011, 2018). Recent research on the development of CSA and CT in preschool aged children suggests that CT peaks around 1 to 2 years, compared with CSA that continues to increase into late childhood. As a result, the changes in CV during the early years may be largely driven by CSA rather than CT (Lyall et al., 2015). Our findings suggest that CSA may be more sensitive to the effects of PAE during this early developmental period compared with CT, where differences may rather manifest later in childhood and adolescence. This finding may have important implications in terms of how CSA development relates to developmental functioning in children with FASD. Altered CSA structure in children with FASD may have long‐term implications in terms of brain development and cognitive functioning.

There was no PAE by child sex interaction effect on CSA, CT, and CV on the ROIs selected for this study. However, we did note overall child sex differences on total CSA and CV within this cohort that were present before and after adjusting for covariates. Findings are consistent with extensive literature that demonstrates larger brain volumes in boys compared to girls (Kaczkurkin et al., 2019). These sex differences were, however, not evident after accounting for multiple comparisons. The multivariate main effects for sex on our a priori selected CT ROIs were only evident after accounting for our covariates of interest and these findings did not survive multiple comparison correction.

Unlike many studies within FASD with limited information on maternal demographic characteristics that may contribute to outcomes, this study was conducted on a well‐characterized sample. Although our findings demonstrated the effects of PAE after accounting for multiple maternal and sociodemographic risk factors, the study population and context are an important consideration when interpreting these findings. Risk factors apparent in low socioeconomic environments are known to play a key role in brain and neurocognitive development (Hackman & Farah, 2009). It is important to highlight that children within this cohort are exposed to a multitude of interacting risk factors that may interact with PAE and result in complex alterations to their developmental trajectory. Exposure to alcohol in utero, for example, may exacerbate the negative effects of other social risk factors prominent in low SES living contexts (Uban et al., 2020). These findings highlight the need for longitudinal research on samples living in contexts with multiple risk factors.

This study has several limitations to consider. First, this study analyzed cross‐sectional data and is therefore unable to provide insight into the longitudinal trajectory of PAE over development. We did not conduct longitudinal comparisons with the neonate imaging data as only a subgroup of those scanned at 2 to 3 years of age were also scanned as neonates. Follow‐up longitudinal analyses of data within this cohort will be required to establish how the developmental trajectory of subcortical and cortical ROIs differs by alcohol exposure status. For example, longitudinal studies can identify the extent to which PAE effects persist in specific ROIs. Second, this study analyzed structural MRI data and is unable to provide any conclusions on how connectivity within the sensorimotor and frontoparietal networks were affected by exposure to PAE or by child sex. Follow‐up analyses of functional MRI data will be valuable in identifying how PAE influences functional brain networks during this period. Investigating functional connectivity patterns in the ROIs identified in our study will provide valuable insight into how structural outcomes relate to functional development in this sample. Third, these data were collected before children were formally assessed for dysmorphology associated with FASD, it is thus not possible to differentiate PAE effects related to specific diagnoses. Our findings, however, do contribute to the literature that identifies regions that may be most vulnerable to the effects of alcohol exposure. From a clinical perspective, our findings show that structural gray matter alterations in children at‐risk for FASD can be identified early in development. From a research perspective, identifying regions implicated by PAE early in development is critical to address the relationship between structural abnormalities identified early in development and debilitating functional outcomes that become apparent later in childhood, given that there is a lack of data on the effects of PAE during early childhood. Fourth, challenges with conducting imaging on young children are numerous and missing data was inevitable. Prior work on this cohort identified similar sociodemographic profiles between those with and without successful scans (Wedderburn et al., 2020). There does, however, remain the possibility that scan success was impacted by alcohol exposure status. In addition, missing data due to exclusions (e.g., failed processing and poor scan quality ), extreme outliers, and missing demographic data may limit the generalizability of these findings. Replication is necessary to determine the extent to which these findings are impacted by biases in the sample. Finally, examining multiple ROIs was important to identify the impact of PAE on specific brain regions during this developmental period. However, larger samples are required to corroborate these findings. To optimize the likelihood of obtaining sufficient samples for analyses, future research should consider employing real‐time prospective motion correction and scan quality evaluation to mitigate against data loss due to motion artifacts. In addition, larger samples and use of a longitudinal design may also allow for a thorough investigation of the potential moderating or mediating roles the covariates (in particular maternal depression, smoking, and nutrition in addition to SES) play in the impact of PAE and child sex on brain development. Given that many children with PAE develop in contexts with prevalent poverty, the authors recommend that future studies obtain data on prenatal and childhood socioeconomic circumstances. Identifying the extent to which SES may buffer the effects of PAE on the development of boys and girls may be an important way to differentiate risk of poor developmental outcomes among those with FASD.

## CONCLUSIONS

In conclusion, this study is one of the first to investigate the effects of PAE and child sex on structural gray matter outcomes at 2 to 3 years of age. Our findings on the effects of alcohol exposure were robust after accounting for the multiple confounds prevalent in this sample and among populations vulnerable to FASD. Our study sheds light on the structural outcomes associated with PAE during a period of development during which the effects of PAE are poorly understood. We contribute to a growing literature on the widespread impact of PAE on subcortical and cortical gray matter development, describing persistence of gray matter alterations identified in the first weeks of life as well as additional findings on the effect of PAE on development within the parietal lobe. Our findings highlight the need for research to consider the differential impact of PAE on male children and female children. Furthermore, findings can inform further research that identifies how ROIs affected by in utero alcohol exposure change over the developmental period.

## CONFLICTS OF INTEREST

The authors declare no conflict of interest with respect to the work in this manuscript.

## Supporting information


Table S1‐S3
