# Supplementary material for: The impact of prenatal alcohol exposure on gray matter volume and cortical surface area of 2 to 3‐year‐old children in a South African birth cohort
Source: Alcohol Clin Exp Res. 2022 May 29;46(7):1233–47. doi: 10.1111/acer.14873 (PMC9357164; doi:10.1111/acer.14873)
Supplement: Supplementary file 1 — Table S1‐S3 [file ACER-46-1233-s001.docx]

| Supplementary Table 1. Multi- and follow up univariate effects for subcortical volume, CSA, CV, and CT unadjusted for covariates | | | | | |
| --- | --- | --- | --- | --- | --- |
| Multivariate Effects | | Pillai’s Trace | F value | Sig. | Partial Eta Squared |
| Subcortical volume (cm^3^) | |  |  |  |  |
| PAE effect | | .13 | 1.46 | .14 | .13 |
| Child sex effect | | .23 | 2.94 | < .001 | .23 |
| PAE X child sex interaction effect | | .16 | 1.94 | .03 | .16 |
| CSA (cm^2^) | |  |  |  |  |
| PAE effect | | .21 | 2.19 | .01 | .21 |
| Child sex effect | | .23 | 2.43 | .002 | .23 |
| PAE X child sex interaction effect | | .11 | 1.05 | .41 | .11 |
| CV (cm^3^) | |  |  |  |  |
| PAE effect | | .23 | 2.44 | .002 | .23 |
| Child sex effect | | .24 | 2.66 | < .001 | .24 |
| PAE X child sex interaction effect | | .10 | 0.89 | .59 | .10 |
| CT (mm) | |  |  |  |  |
| PAE effect | | .11 | 1.03 | .43 | .11 |
| Child sex effect | | .10 | 0.90 | .59 | .10 |
| PAE X child sex interaction effect | | .12 | 1.17 | .29 | .12 |
| Univariate Effect | ROI | Hemisphere | F value | Sig. | Partial Eta Squared |
| Subcortical Volume ROIs (cm^3^) |  |  |  |  |  |
| Child sex effect |  |  |  |  |  |
|  | Amygdala | L | **21.75** | **< .001** | **.13** |
|  | Caudate | L | **4.25** | **.04** | **.03** |
|  | Hippocampus | L | **21.27** | **< .001** | **.12** |
|  | Nucleus accumbens | L | **13.61** | **< .001** | **.08** |
|  | Pallidum | L | **3.22** | **.08** | **.02** |
|  | Putamen | L | **3.27** | **.07** | **.02** |
|  | Thalamus | L | **19.25** | **< .001** | **.11** |
|  | Amygdala | R | **21.52** | **< .001** | **.13** |
|  | Caudate | R | **4.69** | **.03** | **.03** |
|  | Hippocampus | R | **17.61** | **< .001** | **.10** |
|  | Nucleus accumbens | R | **13.55** | **< .001** | **.08** |
|  | Pallidum | R | **2.43** | **.12** | **.02** |
|  | Putamen | R | **2.38** | **.13** | **.02** |
|  | Thalamus | R | **18.31** | **< .001** | **.11** |
| PAE X child sex interaction effect |  |  |  |  |  |
|  | Amygdala | L | 0.79 | .38 | .01 |
|  | Caudate | L | 0.12 | .73 | .001 |
|  | Hippocampus | L | 6.75 | .01 | .04 |
|  | Nucleus accumbens | L | 0.35 | .55 | .002 |
|  | Pallidum | L | 0.67 | .42 | .004 |
|  | Putamen | L | 2.11 | .15 | .01 |
|  | Thalamus | L | 0.88 | .35 | .01 |
|  | Amygdala | R | 2.25 | .14 | .02 |
|  | Caudate | R | 0.01 | .93 | < .001 |
|  | Hippocampus | R | 7.30 | .01 | .05 |
|  | Nucleus accumbens | R | 0.004 | .95 | < .001 |
|  | Pallidum | R | 0.05 | .83 | < .001 |
|  | Putamen | R | 2.13 | .15 | .01 |
|  | Thalamus | R | 1.51 | .22 | .01 |
|  |  |  |  |  |  |
| CSA ROIs (cm^2^) |  |  |  |  |  |
| Child sex effect |  |  |  |  |  |
|  | Anterior cingulate (Caudal) | L | **6.73** | **.01** | **.04** |
|  | Anterior cingulate (Rostral) | L | **10.36** | **.002** | **.06** |
|  | Inferior parietal | L | **11.71** | **< .001** | **.07** |
|  | Middle frontal (Caudal) | L | **20.25** | **< .001** | **.11** |
|  | Middle frontal (Rostral) | L | **15.62** | **< .001** | **.09** |
|  | Paracentral | L | **10.36** | **.002** | **.06** |
|  | Postcentral | L | **15.91** | **< .001** | **.09** |
|  | Precentral | L | **32.62** | **< .001** | **.16** |
|  | Precuneus | L | **16.08** | **< .001** | **.09** |
|  | Anterior cingulate (Caudal) | R | **12.67** | **< .001** | **.07** |
|  | Anterior cingulate (Rostral) | R | **11.57** | **< .001** | **.07** |
|  | Inferior parietal | R | **8.85** | **.003** | **.05** |
|  | Middle frontal (Caudal) | R | **11.15** | **.001** | **.06** |
|  | Middle frontal (Rostral) | R | **24.87** | **< .001** | **.13** |
|  | Paracentral | R | **6.75** | **.01** | **.04** |
|  | Postcentral | R | **6.15** | **.01** | **.04** |
|  | Precentral | R | **17.90** | **< .001** | **.10** |
|  | Precuneus | R | **13.17** | **< .001** | **.07** |
|  |  |  |  |  |  |
| PAE effect |  |  |  |  |  |
|  | Anterior cingulate (Caudal) | L | 2.81 | .10 | .02 |
|  | Anterior cingulate (Rostral) | L | **11.92** | **< .001** | **.07** |
|  | Inferior parietal | L | **19.68** | **< .001** | **.11** |
|  | Middle frontal (Caudal) | L | **7.32** | **.01** | **.04** |
|  | Middle frontal (Rostral) | L | 1.60 | .21 | .01 |
|  | Paracentral | L | **11.56** | **< .001** | **.07** |
|  | Postcentral | L | **12.81** | **< .001** | **.07** |
|  | Precentral | L | **8.57** | **.004** | **.05** |
|  | Precuneus | L | **10.10** | **.002** | **.06** |
|  | Anterior cingulate (Caudal) | R | 1.08 | .30 | .01 |
|  | Anterior cingulate (Rostral) | R | **7.93** | **.01** | **.05** |
|  | Inferior parietal | R | 9.02 | .003 | .05 |
|  | Middle frontal (Caudal) | R | 2.48 | .12 | .02 |
|  | Middle frontal (Rostral) | R | 1.21 | .27 | .01 |
|  | Paracentral | R | **12.37** | **< .001** | **.07** |
|  | Postcentral | R | **12.60** | **< .001** | **.07** |
|  | Precentral | R | **4.55** | **.03** | **.03** |
|  | Precuneus | R | **12.75** | **.001** | **.07** |
|  |  |  |  |  |  |
| CV ROIs (cm^3^) |  |  |  |  |  |
| Child Sex effect |  |  |  |  |  |
|  | Anterior cingulate (Caudal) | L | 1.48 | .23 | .01 |
|  | Anterior cingulate (Rostral) | L | **10.16** | **.002** | **.06** |
|  | Inferior parietal | L | **14.63** | **< .001** | **.08** |
|  | Middle frontal (Caudal) | L | **13.61** | **< .001** | **.08** |
|  | Middle frontal (Rostral) | L | **11.40** | **< .001** | **.06** |
|  | Paracentral | L | **8.53** | **.004** | **.05** |
|  | Postcentral | L | **22.87** | **< .001** | **.12** |
|  | Precentral | L | **30.08** | **< .001** | **.15** |
|  | Precuneus | L | **13.41** | **< .001** | **.07** |
|  | Anterior cingulate (Caudal) | R | **8.88** | **.003** | **.05** |
|  | Anterior cingulate (Rostral) | R | 3.70 | .06 | .02 |
|  | Inferior parietal | R | **8.63** | **.004** | **.05** |
|  | Middle frontal (Caudal) | R | **24.18** | **< .001** | **.13** |
|  | Middle frontal (Rostral) | R | **16.50** | **< .001** | **.09** |
|  | Paracentral | R | 2.71 | .10 | .02 |
|  | Postcentral | R | **12.21** | **< .001** | **.07** |
|  | Precentral | R | **14.03** | **< .001** | **.08** |
|  | Precuneus | R | **8.40** | **.004** | **.05** |
|  |  |  |  |  |  |
| PAE effect |  |  |  |  |  |
|  | Anterior cingulate (Caudal) | L | 1.19 | .28 | .01 |
|  | Anterior cingulate (Rostral) | L | **13.17** | **< .001** | **.07** |
|  | Inferior parietal | L | **9.45** | **.002** | **.05** |
|  | Middle frontal (Caudal) | L | 3.61 | .06 | .02 |
|  | Middle frontal (Rostral) | L | **16.94** | **< .001** | **.09** |
|  | Paracentral | L | **10.54** | **.001** | **.06** |
|  | Postcentral | L | **11.36** | **< .001** | **.06** |
|  | Precentral | L | **12.26** | **< .001** | **.07** |
|  | Precuneus | L | **10.30** | **.002** | **.06** |
|  | Anterior cingulate (Caudal) | R | 0.71 | .40 | .004 |
|  | Anterior cingulate (Rostral) | R | **6.96** | **.01** | **.04** |
|  | Inferior parietal | R | 3.26 | .07 | .02 |
|  | Middle frontal (Caudal) | R | 1.58 | .21 | .01 |
|  | Middle frontal (Rostral) | R | 4.22 | .04 | .03 |
|  | Paracentral | R | **7.87** | **.01** | **.05** |
|  | Postcentral | R | **7.35** | **.01** | **.04** |
|  | Precentral | R | **7.67** | **.01** | **.04** |
|  | Precuneus | R | **11.28** | **< .001** | **.06** |
| Note. Follow-up univariate ANOVA tests were conducted following significant multivariate findings. CSA = Cortical surface area, CV = Cortical volume, CT = Cortical thickness. Findings that survive FDR correction indicated in bold. | | | | | |

| Supplementary Table 2. Multi- and follow up univariate effects for subcortical volume, CSA, CV, and CT, adjusted for ICV | | | | | |
| --- | --- | --- | --- | --- | --- |
| Multivariate Effects | | Pillai’s Trace | F value | Sig. | Partial Eta Squared |
| Subcortical volume (cm^3^) | |  |  |  |  |
| PAE effect | | .13 | 1.44 | .14 | .13 |
| Child sex effect | | .10 | 1.12 | .34 | .10 |
| PAE X child sex interaction effect | | .16 | 1.82 | .04 | .16 |
| CSA (cm^2^) | |  |  |  |  |
| PAE effect | | .18 | 1.81 | .03 | .18 |
| Child sex effect | | .09 | 0.79 | .71 | .09 |
| PAE X child sex interaction effect | | .11 | 1.02 | .44 | .11 |
| CV (cm^3^) | |  |  |  |  |
| PAE effect | | .20 | 2.09 | .01 | .20 |
| Child sex effect | | .12 | 1.11 | .35 | .12 |
| PAE X child sex interaction effect | | .09 | 0.84 | .65 | .09 |
| CT (mm) | |  |  |  |  |
| PAE effect | | .12 | 1.15 | .31 | .12 |
| Child sex effect | | .14 | 1.31 | .19 | .14 |
| PAE X child sex interaction effect | | .13 | 1.18 | .28 | .13 |
| Univariate Effect | ROI | Hemisphere | F value | Sig. | Partial Eta Squared |
| Subcortical Volume ROIs (cm3) |  |  |  |  |  |
| PAE X Child sex effect |  |  |  |  |  |
|  | Amygdala | L | 0.11 | .74 | .001 |
|  | Caudate | L | 0.11 | .74 | .001 |
|  | Hippocampus | L | 5.07 | .03 | .03 |
|  | Nucleus accumbens | L | 0.06 | .81 | < .001 |
|  | Pallidum | L | 2.93 | .09 | .02 |
|  | Putamen | L | 4.80 | .03 | .03 |
|  | Thalamus | L | 0.04 | .84 | < .001 |
|  | Amygdala | R | 1.00 | .32 | .01 |
|  | Caudate | R | 0.73 | .39 | .01 |
|  | Hippocampus | R | 5.61 | .02 | .04 |
|  | Nucleus accumbens | R | 0.23 | .64 | .002 |
|  | Pallidum | R | 0.14 | .71 | .001 |
|  | Putamen | R | 4.40 | .04 | .03 |
|  | Thalamus | R | 0.30 | .58 | .002 |
|  |  |  |  |  |  |
| CSA ROIs (cm^2^) |  |  |  |  |  |
| PAE effect |  |  |  |  |  |
|  | Anterior cingulate (Caudal) | L | 0.05 | .82 | < .001 |
|  | Anterior cingulate (Rostral) | L | 4.80 | .03 | .03 |
|  | **Inferior parietal** | **L** | **11.80** | **< .001** | **.07** |
|  | Middle frontal (Caudal) | L | 2.81 | .10 | .02 |
|  | Middle frontal (Rostral) | L | 0.29 | .59 | .002 |
|  | Paracentral | L | 4.98 | .03 | .03 |
|  | Postcentral | L | 5.91 | .02 | .03 |
|  | Precentral | L | 2.28 | .13 | .01 |
|  | Precuneus | L | 3.24 | .07 | .02 |
|  | Anterior cingulate (Caudal) | R | 0.31 | .58 | .002 |
|  | Anterior cingulate (Rostral) | R | 2.08 | .15 | .01 |
|  | Inferior parietal | R | 3.26 | .07 | .02 |
|  | Middle frontal (Caudal) | R | 0.18 | .67 | .001 |
|  | Middle frontal (Rostral) | R | 0.24 | .62 | .001 |
|  | Paracentral | R | 5.85 | .02 | .03 |
|  | Postcentral | R | 6.43 | .01 | .04 |
|  | Precentral | R | 0.41 | .52 | .002 |
|  | Precuneus | R | 5.38 | .02 | .03 |
|  |  |  |  |  |  |
| CV ROIs (cm^3^) |  |  |  |  |  |
| PAE effect |  |  |  |  |  |
|  | Anterior cingulate (Caudal) | L | 0.01 | .92 | .001 |
|  | Anterior cingulate (Rostral) | L | 6.62 | .01 | .04 |
|  | Inferior parietal | L | 5.48 | .02 | .03 |
|  | Middle frontal (Caudal) | L | 0.17 | .68 | .001 |
|  | Middle frontal (Rostral) | L | 9.37 | .003 | .05 |
|  | Paracentral | L | 4.91 | .03 | .03 |
|  | Postcentral | L | 5.63 | .02 | .03 |
|  | Precentral | L | 5.95 | .02 | .04 |
|  | Precuneus | L | 3.54 | .06 | .02 |
|  | Anterior cingulate (Caudal) | R | 0.08 | .79 | < .001 |
|  | Anterior cingulate (Rostral) | R | 2.66 | .11 | .02 |
|  | Inferior parietal | R | 0.56 | .46 | .003 |
|  | Middle frontal (Caudal) | R | 0.01 | .93 | < .001 |
|  | Middle frontal (Rostral) | R | 0.79 | .38 | .01 |
|  | Paracentral | R | 3.59 | .06 | .02 |
|  | Postcentral | R | 2.93 | .09 | .02 |
|  | Precentral | R | 2.60 | .11 | .02 |
|  | Precuneus | R | 4.30 | .04 | .03 |
| Note. Follow-up univariate ANOVA tests were conducted following significant multivariate findings. CSA = Cortical surface area, CV = Cortical volume, CT = Cortical thickness, ICV = Intracranial volume. Findings that survived FDR corrected indicated in bold. | | | | | |

.

| Supplementary Table 3. Multi- and follow up univariate effects for subcortical volume, CSA, CV, and CT adjusted for age, ICV, maternal smoking status, antenatal maternal depression, and maternal education | | | | | |
| --- | --- | --- | --- | --- | --- |
| Multivariate Effects | | Pillai’s Trace | F value | Sig. | Partial Eta Squared |
| Subcortical volume (cm^3^) | |  |  |  |  |
| PAE effect | | .16 | 1.46 | .14 | .16 |
| Child sex effect | | .13 | 1.07 | .39 | .13 |
| PAE X child sex interaction effect | | .19 | 1.81 | .05 | .19 |
| CSA (cm^2^) | |  |  |  |  |
| PAE effect | | .20 | 1.61 | .07 | .20 |
| Child sex effect | | .12 | 0.88 | .61 | .12 |
| PAE X child sex interaction effect | | .15 | 1.08 | .38 | .15 |
| CV (cm^3^) | |  |  |  |  |
| PAE effect | | .22 | 1.79 | .03 | .22 |
| Child sex effect | | .16 | 1.20 | .27 | .16 |
| PAE X child sex interaction effect | | .11 | 0.76 | .74 | .11 |
| CT (mm) | |  |  |  |  |
| PAE effect | | .14 | 0.99 | .47 | .14 |
| Child sex effect | | .23 | 1.87 | .03 | .23 |
| PAE X child sex interaction effect | | .16 | 1.23 | .25 | .16 |
| Univariate Effect | ROI | Hemisphere | F value | Sig. | Partial Eta Squared |
| Subcortical Volume ROIs (cm^3^) |  |  |  |  |  |
| PAE X child sex interaction effect |  |  |  |  |  |
|  | Amygdala | L | 0.05 | .83 | < .001 |
|  | Caudate | L | 0.01 | .93 | < .001 |
|  | Hippocampus | L | 2.49 | .12 | .02 |
|  | Nucleus accumbens | L | 0.05 | .83 | < .001 |
|  | Pallidum | L | 1.13 | .29 | .01 |
|  | Putamen | L | 5.28 | .02 | .04 |
|  | Thalamus | L | 0.74 | .39 | .01 |
|  | Amygdala | R | 0.28 | .60 | .002 |
|  | Caudate | R | 0.13 | .72 | .001 |
|  | Hippocampus | R | 3.21 | .08 | .03 |
|  | Nucleus accumbens | R | 0.51 | .48 | .004 |
|  | Pallidum | R | 0.00 | .99 | < .001 |
|  | Putamen | R | 8.29 | .01 | .07 |
|  | Thalamus | R | 0.66 | .42 | .01 |
|  |  |  |  |  |  |
| CSA ROIs (cm^2^) |  |  |  |  |  |
| PAE effect |  |  |  |  |  |
|  | Anterior cingulate (Caudal) | L | 3.52 | .06 | .03 |
|  | Anterior cingulate (Rostral) | L | 7.08 | .01 | .05 |
|  | **Inferior parietal** | **L** | **11.46** | **< .001** | **.08** |
|  | Middle frontal (Caudal) | L | 3.52 | .06 | .03 |
|  | Middle frontal (Rostral) | L | 0.01 | .91 | < .001 |
|  | Paracentral | L | 4.28 | .04 | .03 |
|  | Postcentral | L | 0.89 | .35 | .01 |
|  | Precentral | L | 1.56 | .21 | .01 |
|  | Precuneus | L | 2.39 | .13 | .02 |
|  | Anterior cingulate (Caudal) | R | 0.01 | .93 | < .001 |
|  | Anterior cingulate (Rostral) | R | 2.10 | .15 | .02 |
|  | Inferior parietal | R | 4.11 | .05 | .03 |
|  | Middle frontal (Caudal) | R | 0.37 | .54 | .003 |
|  | Middle frontal (Rostral) | R | 0.30 | .59 | .002 |
|  | Paracentral | R | 4.61 | .03 | .03 |
|  | Postcentral | R | 3.89 | .05 | .03 |
|  | Precentral | R | 1.24 | .27 | .01 |
|  | Precuneus | R | 4.60 | .03 | .03 |
|  |  |  |  |  |  |
| CV ROIs (cm^3^) |  |  |  |  |  |
| PAE effect |  |  |  |  |  |
|  | Anterior cingulate (Caudal) | L | 0.14 | .71 | .001 |
|  | Anterior cingulate (Rostral) | L | 6.61 | .01 | .05 |
|  | Inferior parietal | L | 7.43 | .01 | .05 |
|  | Middle frontal (Caudal) | L | 6.46 | .01 | .05 |
|  | Middle frontal (Rostral) | L | 0.25 | .62 | .002 |
|  | Paracentral | L | 3.78 | .05 | .03 |
|  | Postcentral | L | 1.13 | .29 | .01 |
|  | Precentral | L | 3.54 | .06 | .03 |
|  | Precuneus | L | 2.32 | .13 | .02 |
|  | Anterior cingulate (Caudal) | R | 0.03 | .87 | < .001 |
|  | Anterior cingulate (Rostral) | R | 3.39 | .07 | .03 |
|  | Inferior parietal | R | 1.80 | .18 | .01 |
|  | Middle frontal (Caudal) | R | 0.90 | .35 | .01 |
|  | Middle frontal (Rostral) | R | 0.10 | .75 | .001 |
|  | Paracentral | R | 2.86 | .09 | .02 |
|  | Postcentral | R | 1.67 | .20 | .01 |
|  | Precentral | R | 3.12 | .08 | .02 |
|  | Precuneus | R | 3.48 | .06 | .03 |
|  |  |  |  |  |  |
| CT ROIs (mm) |  |  |  |  |  |
| Child sex effect |  |  |  |  |  |
|  | Anterior cingulate (Caudal) | L | 0.02 | .90 | < .001 |
|  | Anterior cingulate (Rostral) | L | 1.55 | .22 | .01 |
|  | Inferior parietal | L | 0.26 | .61 | .002 |
|  | Middle frontal (Caudal) | L | 0.02 | .89 | <.001 |
|  | Middle frontal (Rostral) | L | 0.20 | .65 | .002 |
|  | Paracentral | L | 0.74 | .39 | .01 |
|  | Postcentral | L | 5.92 | .02 | .04 |
|  | Precentral | L | 0.08 | .79 | .001 |
|  | Precuneus | L | 0.83 | .36 | .01 |
|  | Anterior cingulate (Caudal) | R | 0.62 | .43 | .01 |
|  | Anterior cingulate (Rostral) | R | 0.44 | .51 | .003 |
|  | Inferior parietal | R | 6.31 | .01 | .05 |
|  | Middle frontal (Caudal) | R | 0.12 | .73 | .001 |
|  | Middle frontal (Rostral) | R | 0.69 | .41 | .01 |
|  | Paracentral | R | 2.12 | .15 | .02 |
|  | Postcentral | R | 4.40 | .04 | .03 |
|  | Precentral | R | 0.08 | .78 | .001 |
|  | Precuneus | R | 0.70 | .41 | .01 |
| Note. Follow-up univariate ANOVA tests were conducted following significant multivariate findings. CSA = Cortical surface area, CV = Cortical volume, CT = Cortical thickness, ICV = Intracranial volume. Findings that survived FDR corrected indicated in bold. | | | | | |
